# Changes in *PTGS1* and *ALOX12* Gene Expression in Peripheral Blood Mononuclear Cells Are Associated with Changes in Arachidonic Acid, Oxylipins, and Oxylipin/Fatty Acid Ratios in Response to Omega-3 Fatty Acid Supplementation

**DOI:** 10.1371/journal.pone.0144996

**Published:** 2015-12-16

**Authors:** Claire C. Berthelot, Shizuo George Kamita, Romina Sacchi, Jun Yang, Malin L. Nording, Katrin Georgi, Christine Hegedus Karbowski, J. Bruce German, Robert H. Weiss, Ronald J. Hogg, Bruce D. Hammock, Angela M. Zivkovic

**Affiliations:** 1 University of Picardie Jules Verne, Amiens - Somme, France; 2 University of Technology in Compiègne, Compiègne—Oise, France; 3 Department of Nutrition, University of California Davis, Davis, CA, United States Of America; 4 Department of Entomology, University of California Davis, Davis, CA, United States Of America; 5 Department of Chemistry, Umeå University, Umeå, Sweden; 6 Comprehensive Cancer Center, University of California Davis, Davis, CA, United States Of America; 7 Department of Food Science & Technology, University of California Davis, Davis, CA, United States Of America; 8 Foods for Health Institute, University of California Davis, Davis, CA, United States Of America; 9 Nephrology Division, Department of Medicine, University of California Davis, Davis, CA, United States Of America; 10 Medical Service, Sacramento VA Medical Center, Sacramento, CA, United States Of America; 11 Scott & White Clinic, Temple, TX, United States Of America; University of Milan, ITALY

## Abstract

**Introduction:**

There is a high degree of inter-individual variability among people in response to intervention with omega-3 fatty acids (FA), which may partly explain conflicting results on the effectiveness of omega-3 FA for the treatment and prevention of chronic inflammatory diseases. In this study we sought to evaluate whether part of this inter-individual variability in response is related to the regulation of key oxylipin metabolic genes in circulating peripheral blood mononuclear cells (PBMCs).

**Methods:**

Plasma FA and oxylipin profiles from 12 healthy individuals were compared to PBMC gene expression profiles following six weeks of supplementation with fish oil, which delivered 1.9 g/d eicosapentaenoic acid (EPA) and 1.5 g/d docosahexaenoic acid (DHA). Fold changes in gene expression were measured by a quantitative polymerase chain reaction (qPCR).

**Results:**

Healthy individuals supplemented with omega-3 FA had differential responses in prostaglandin-endoperoxide synthase 1 (*PTGS1*), prostaglandin-endoperoxide synthase 2 (*PTGS2*), arachidonate 12-lipoxygenase (*ALOX12*), and interleukin 8 (*IL-8*) gene expression in isolated PBMCs. In those individuals for whom plasma arachidonic acid (ARA) in the phosphatidylethanolamine (PE) lipid class decreased in response to omega-3 intervention, there was a corresponding decrease in gene expression for *PTGS1* and *ALOX12*. Several oxylipin product/FA precursor ratios (e.g. prostaglandin E_2_ (PGE_2_)/ARA for *PTGS1* and 12-hydroxyeicosatetraenoic acid (12-HETE)/ARA for *ALOX12*) were also associated with fold change in gene expression, suggesting an association between enzyme activity and gene expression. The fold-change in *PTGS1* gene expression was highly positively correlated with *ALOX12* gene expression but not with *PTGS2*, whereas *IL-8* and *PTGS2* were positively correlated.

**Conclusions:**

The regulation of important oxylipin metabolic genes in PBMCs varied with the extent of change in ARA concentrations in the case of *PTGS1* and *ALOX12* regulation. PBMC gene expression changes in response to omega-3 supplementation varied among healthy individuals, and were associated with changes in plasma FA and oxylipin composition to different degrees in different individuals.

**Trial Registration:**

clinicaltrials.gov NCT01838239

## Introduction

Omega-3 fatty acids (FA) work as therapeutic agents by a wide range of biological mechanisms leading to anti-inflammatory and immune modulating effects. Most individuals consuming a Western diet are likely to be effectively deficient in long-chain omega-3 FA since the Western diet is heavily skewed toward omega-6 FA. Globally, humans currently consume an omega-6:omega-3 ratio closer to 15:1, which is dramatically shifted from the historical ratio of 2:1[1]. Omega-3 and omega-6 FA are essential because humans lack the specific desaturase enzyme required for their production. The polyunsatured fatty acids (PUFA) can be converted into oxylipins by interaction with reactive oxygen and three main classes of enzymes including cyclooxygenase (COX), lipoxygenase (LOX), and cytochrome P450 (CYP) [2]. The COX pathway generates several pro-coagulant and proliferative metabolites from arachidonic acid (ARA) including prostaglandins (e.g. PGE_2_) and thromboxanes (e.g. TXB_2_). The EPA COX products PGD_3_ and PGE_3_ are less proliferative than their ARA counterparts, whereas Resolvin E1 is pro-resolving (i.e. leads to cessation of inflammation). Pro-inflammatory products are produced from ARA via the 5-LOX pathway (e.g. 5-hydroxyeicosatetraenoic acid (5-HETE)). This pathway also generates hydroxypentaenoic acids (HEPEs) from EPA. Some other pro-inflammatory products come from the 12- and 15-LOX pathways such as 9-HETE, 11-HETE, 12-HETE, 15-HETE, and metabolites from linoleic acid (LA). In fact over 90 bioactive oxylipins are produced from omega-3 and omega-6 FA. These oxylipins form a complex mixture that induces both pro- and anti- inflammatory effects depending on the site of action, the cell from which they are produced, and the stage of inflammation. In general the omega-3 oxylipins are antagonistic to the actions of the omega-6 oxylipins [3] and are thus considered to be anti-inflammatory and pro-resolving.

The latest research on omega-3 FA and their beneficial effects in diseases such as cardiovascular disease have been inconsistent, with large clinical trials reporting benefit, no benefit, and even detrimental effects in some cases. In fact, recent meta-analyses have found the literature inconsistent enough to call into question the recommendation to use omega-3 FA for the treatment and prevention of inflammatory diseases such as heart disease [4]. Recently, we showed that this observed inconsistency in the literature on the effects of omega-3 FA may be due in part to the high degree of inter-individual variability among people in response to intervention with omega-3 FA[5]. We found that while certain aspects of response to intervention were universal across all of the subjects, the magnitude and sometimes both the magnitude and direction of response in both FA and oxylipin profiles were highly variable.

The aim of this pilot study was to determine whether the observed inter-individual variability in response to omega-3 FA intervention may be due to variability in the regulation of gene transcription of key genes involved in the production of inflammatory lipid mediators. The primary outcomes were correlations among the fold changes in gene expression in circulating immune cells and percentage changes in plasma FA and oxylipins.

## Materials and Methods

### Clinical protocol

All of the clinical study parameters are described in Nording et al. [5]. The study was an open label and single arm intervention study. Recruitment took place on the University of California Davis campus and included posting of flyers as well as announcements at seminars. Eligibility criteria included age 18–65, not being pregnant or nursing, not having any existing conditions or diseases, BMI 18–30, not having anemia and/or other conditions affecting ability to donate blood safely, not recently recovering from illnesses, injuries, or infections, able and willing to take 6 g of fish oil every day for 6 weeks, able to stop or avoid taking NSAID and other anti-inflammatory medications (e.g. allergy medications) and able and willing to avoid consuming seafood for 6 weeks.

Subjects came to the study site (the Ragle Human Nutrition Center on the University of California Davis campus) three times. At the first visit subjects were screened and filled out questionnaires about their health and medications, and height and weight was measured to assess their eligibility. If subjects met eligibility criteria they were consented to participate in the study. At the second (i.e. baseline or pre intervention) and third (i.e. post intervention) visits subjects came to the study center in the morning after an overnight fast. Blood samples were collected by venipuncture using BD Vacutainer lavender-top EDTA tubes, and height and weight were measured. Within 15 minutes of collection whole blood was centrifuged in a tabletop centrifuge (10 minutes, 4°C, 13,000 rpm) and the plasma was immediately aliquoted and frozen at -70°C until they were analyzed. At the baseline or pre intervention visit, subjects received their intervention, which consisted of 6 individual bottles each containing a week’s worth of fish oil capsules. Subjects received instructions to take 6 1-gram capsules each day and to continue consuming their regular diets but to abstain from consuming any seafood or taking any other fish oil supplements. The fish oil capsules totaled 1.9 g/d EPA and 1.5 g/d DHA each day, and were from Ocean Nutrition (lot 18394). Each capsule was composed of 35% EPA, 26% DHA, 11% 20:1n9, 6% 22:5n3, 6% 22:1n11, 2% 18:1n9, 2% 20:4n6, 2% 20:4n3, with the remainder consisting of other minor FA each at ≤1% concentration. Subjects were contacted throughout the study by e-mail and/or phone by study personnel to encourage compliance.

**Fig 1:**
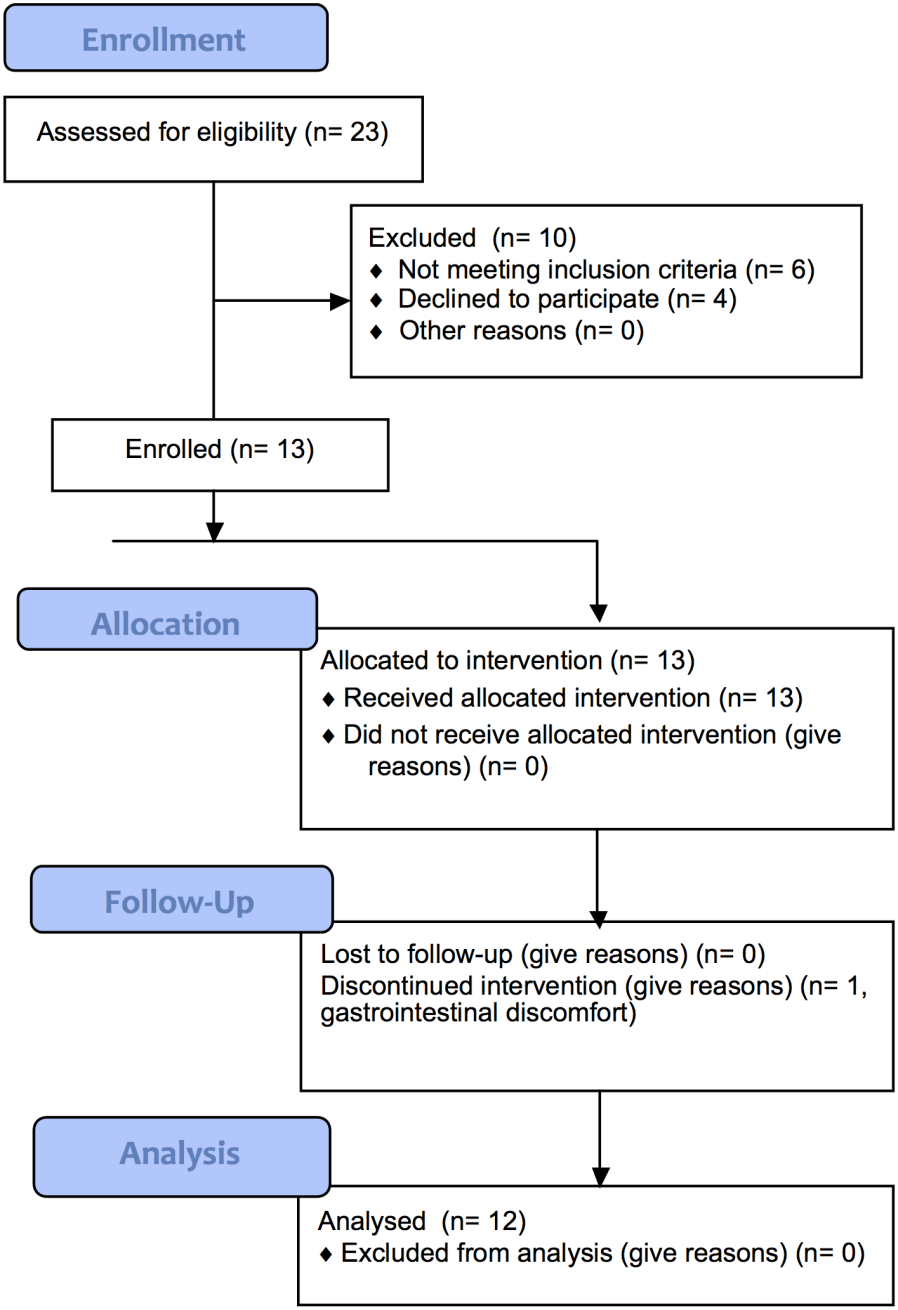
Consort Diagram.

After 6 weeks at the post-intervention visit subjects returned to the study site and another fasting blood draw was taken, and weight and height was measured. Subjects reported their dietary intake with 3-day diet records at the start and end of the study. Subjects were compensated with gift cards and were given their baseline lipid results. The consort diagram (Fig 1) shows that 23 subjects were screened, 10 were excluded (6 were either taking medications or were not willing to abstain from taking medications and 4 declined enrollment), and the remaining 13 were enrolled. One subject dropped from the study after the first day of the intervention due to gastrointestinal discomfort (diarrhea). The remaining 12 subjects all completed the study and were included in analysis, and consisted of 8 female and 4 male subjects. No adverse events were reported however 2 of the female subjects reported that their menstrual cycles were longer than normal during the intervention. The 12 subjects who completed the study had the following mean (± SD) characteristics: age, 32 ± 8 y; weight, 67 ± 17 kg; body mass index (BMI), 23 ± 3 kg/m^2^ (median: 22, range: 18–28) (individual characteristics shown in S1 Table).

The date range for participant recruitment and follow-up was May 03, 2007 through January 18, 2010. This trial was registered at clinicaltrials.gov as NCT01838239. This study was registered after enrollment of participants started due to an unintentional administrative delay. The authors confirm that all ongoing and related trials for this intervention are registered. Sample size calculations were not performed due to the exploratory nature of this study.

### Ethics Statement

This study followed the principles expressed in the Declaration of Helsinki. The experimental protocol was approved by the Institutional Review Board of the University of California Davis on May 03. 2007, and written informed consent was obtained from all of the subjects.

### PBMC isolation and RNA extraction

10 mL of whole anticoagulated blood was centrifuged at 5000*g* at 4°C for 15 min (Beckman Coulter Allegra 6R Centrifuge). Following centrifugation, the plasma layer was removed and the PBMC layer was carefully isolated, transferred to a tube with 2 mL of sterile PBS, and carefully mixed. The PBMC+PBS mixture was gently layered onto Ficoll-Paque (Sigma-Aldrich) and centrifuged at 450*g* for 10 min. After centrifugation, the supernatant was removed and the cells were re-suspended and transferred into 15 mL tubes with 5 mL of PBS and mixed gently prior to a second centrifugation at 450*g* for 10 min. Finally, the cells were re-suspended in 1 mL of PBS and centrifuged at 1000*g* in a micro-centrifuge (Eppendorf Micro Centrifuge 5417R) for 1 min at 4°C. After centrifugation the cells were re-suspended in 400 μL of RNA Later (Ambion), stored overnight at 4°C and then at -80°C until RNA extraction. Total RNA was extracted from 2 x 10^7^ cells using TRIzol Reagent (Life Technologies) in combination with a PureLink RNA Mini Kit (Life Technologies). The concentration and integrity of the RNA samples were determined using an Experion Automated Electrophoresis System (BioRad), and only RNA with a RNA quality indicator (RQI) value greater than 9.2 was used in the cDNA synthesis reaction.

### TaqMan Real-Time RT-PCR

First-strand cDNA was synthesized from 168 ng of total RNA by using a High-Capacity cDNA Reverse Transcription kit (Applied Biosystems) under conditions recommended by the manufacturer. The expression levels of 4 endogenous control genes (Actin, GAPDH, HPRT1, and GUSB) and 92 target genes were quantified using custom pre-designed TaqMan probes and primers in a 96 well-plate format (Applied Biosystems). Primer and probe sequences and quality controls, including gene amplification specificities, were provided by the manufacturer. Briefly, the cDNA amplification was performed on 50-fold diluted cDNA samples using TaqMan Fast Advanced Master Mix (Applied Biosystems), and the following cycling conditions were run on a Prism 7500 Fast real-time PCR thermocycler (Applied Biosystems): 50°C/ 2 min; 95°C/ 20 sec; and 40 cycles of 95°C/ 3sec; 60°C/ 30 sec.

The Pfaffl method [6] was used to calculate the expression levels of the target genes in the post-treatment samples relative to the corresponding baseline control samples within each subject and normalized against HPRT1 and GUSB. Individual amplification efficiencies were analyzed for each reaction by LinRegPCR software (HFRC version 2014.2, [7]) and ΔCt values were corrected by their corresponding individual efficiencies.

### Fatty acid analysis

Fatty acid analysis was performed by Lipomics Technologies Inc. (West Sacramento, CA) according to the method described by Watkins et al. [8]. Coefficients of variation for this method are described in Zivkovic et al. [9]. Briefly, by using a modified Folch extraction [10], the lipids from plasma were extracted and then separated by preparative high-pressure liquid chromatography into seven lipid classes: cholesterol esters (CE), diglycerides (DG), free fatty acids (FFA), lyso phosphatidylcholine (LY), phosphatidylcholine (PC), phosphatidylethanolamine (PE), and triglycerides (TG). Internal standards were used and calibration curves were calculated in order to quantify the fatty acids. FA methyl esters, from all lipid classes, produced by trans-esterification were extracted and analyzed by gas chromatography. All of the FA were quantified (nmol FA/g plasma) in each lipid class and summed in order to obtain total lipid class quantities. Enzyme activity was measured from product/precursor ratios as described in Warensjö et al. [11]. Warensjö et al. showed that 16:1n7/16:0 and 18:1n9/18:0 are highly correlated with the activity of the stearoyl-CoA-desaturase (SCD) enzyme. In this study, the following product/precursor ratios were used as indicators of the activities of *PTGS1* and *PTGS2*: PGE_2_/20:4n6, PGD_2_/20:4n6, PGF_2α_/20:4n6, and TXB_2_/20:4n6; *ALOX 12*: 12-HETE/20:4n6 and 12-HEPE/20:5n3; and *CYP4A11*: 20-HETE/20:4n6.

### Oxylipin profiling

Oxylipins were analyzed as described in Yang et al. [12]. Briefly, samples were extracted by solid phase extraction (SPE) and separated by liquid chromatography, followed by QTrap tandem mass spectrometry. The instrument was operated in negative multiple reaction monitoring (MRM) mode. Quality control samples were analyzed at a minimum frequency of 10 samples. Analyst software 1.4.2 was used to quantify the peaks according to the standard curves. Oxylipin data were quantified and are presented as mol/L of plasma. Six batches of the standard mixtures and three further dilutions were used to determine the Limit of Quantification (LOQ) and linearity range. The calibration curves were calculated by least-squares linear regression using an 1/x weighting factor. The standard concentrations were back-calculated from the constructed calibrations curves for each analyte. Any oxylipins that were below LOQ were excluded from analysis.

### Statistical analysis

The following genes were excluded from the qPCR data analysis because they met one or more of the following exclusion criteria 1) showed undetectable or unreliable expression levels (Ct>35), 2) primers could potentially amplify genomic DNA, 3) amplification efficiencies were outside of the acceptable range (1.85–2.00): *IL1B*, *IL2*, *TNFAIP3*, *TLR1*, *VCAM1*, *TLR8*, *CYP2J2*, *MYD88*, *TNFRSF11A*, *PTGES*, *EPHX3*, *EPHX4*, *TLR3*, *TLR4*, *NFKBIA*, *RELA*, *MYC*, *IRAK1*, *LTBR*, *ICAM1*, *IL1R1*, *IL12A*, *IL10*, *TNF*, *IL6*, *CSF1*, *NFKB2*, *IKBKG*, *IRAK2*, *MAP3K7*, *MAP3K14*, *TBK1*, *BCL3*, *FASLG*, *NFKBIB*, *BTRC*, *TRAF5*, *TRAF2*, *CD83*, *TRAF4*, *TRAF1*, *ALOX5*, *ENPP2*, *CREB1*, *CREBBP*, *CCL2*, *NFKBIE*, *STAT1*, *TRAF3*, *TNFSF11*, *PTAFR*, *TNFRSF10A*, *TLR6*, *PLA2G7*, *TNFS15*, *TNFRSF10B*, *LTA4H*, *TBXAS1*, *TLR9*, *PTGDS*, *MAP3K1*, *IKBKB*, *IRAK1BP1*, *ZNF675*, *BCL2*, *TLR7*, *NFKB1*, *IL2RA*, *BCL10*, *REL*, *EPHX1*, *CHUK*, *TNFRSF1A*, *EPHX2*, *TLR2*, *CD40*, *18S*, *PTAFR*, *TLR6*, *TNFSF15*, *CD14*, *CYP4A11*, *CRP*, *CSF3*, *NOS2*, *CYP2C19*, *SELE*, *IL1A*, *CYP4F2*, *IL12B*, *CARD14*, *PTGIS*, *and CSF3*.

Product/precursor ratios were used as indicators of enzyme activity for *PTGS1*, *PTGS2*, *ALOX12*, and *CYP4A11*. For *PTGS1* and *PTGS2* the following ratios were used: PGE_2_/20:4n6, PGD_2_/20:4n6, PGF_2α_/20:4n6, and TXB_2_/20:4n6. For *ALOX12* the following ratios were used: 12-HETE/20:4n6, and 12-HEPE/20:5n3. For CYP4A11 the ratio 20-HETE/20:4n6 was used. For each of these sets of ratios the FA from each of the 7 lipid classes were examined.

Percentage change for each variable was calculated as $\frac{\left(Post-Pre\right)}{Pre}*100$. The percentage change (% change) in the following variables was analyzed for correlations with fold-change in gene expression using Pearson product-moment correlation analysis: ARA, EPA, DHA, PGE_2_, PGD_2_, PGF_2α_, TXB_2,_ 12-HETE, 12-HEPE, 20-HETE, and the appropriate product/precursor ratios for each gene as described above. Pearson product-moment correlation analysis was also used to examine the correlations between BMI and the fold changes in gene expression and % changes in oxylipins, FA, and oxylipin/FA ratios. Significance was set at α ≤ 0.05. The Fisher transformation was used to evaluate statistical significance and to obtain 95% confidence intervals. No adjustments were made for multiple testing due to the exploratory nature of the analyses. The data were analyzed using JMP12 Pro software (SAS Institute, Inc., Cary, NC).

## Results

**Fig 2:**
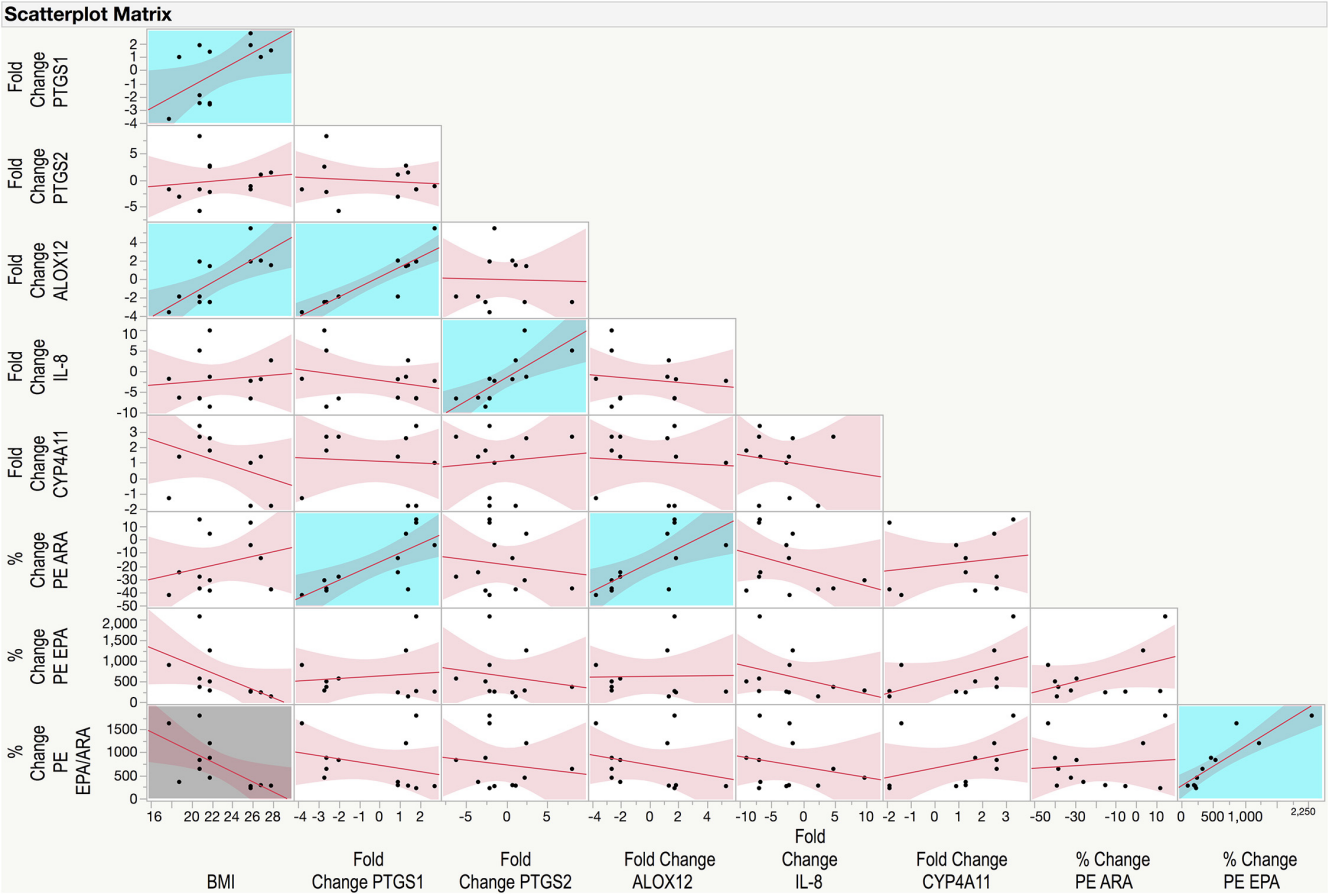
Scatterplot matrix of correlations among body mass index (BMI), fold changes in gene expression of *PTGS1*, *PTGS2*, *ALOX12*, *CYP4A11*, and *IL-8*, and percentage changes in phosphatidylethanolamine (PE) arachidonic acid (ARA, 20:4n6), PE eicosapentaenoic acid (EPA, 20:5n3), and PE EPA/ARA ratio. Correlation analysis was conducted using Pearson product-moment correlation analysis. Statistically significant positive correlations are shown highlighted with a blue background, and negative correlations are shown highlighted with a grey background.

Five genes met all quality control criteria and were included in the subsequent analysis evaluating their relationships with plasma FA and oxylipin profiles: *PTGS1*, *PTGS2*, *ALOX12*, *IL-8*, and *CYP4A11*. The fold change results (mean ± SD) for the 5 included genes were as follows: *PTGS1*–0.14 ± 2.29; *PTGS2*–0.18 ± 3.65; *ALOX12*–0.06 ± 2.77; *IL-8*–2.02 ± 5.58; *CYP4A11* 1.10 ± 1.90. A scatterplot matrix of correlations among the 5 genes as well as BMI, % change in PE ARA, EPA, and EPA/ARA ratio is depicted in Fig 2. Across all 5 genes there was a range of responses from a negative fold change in gene expression to a positive fold change (minimum, maximum): *PTGS1*–3.7, +2.8; *PTGS2*–5.9, +8.3; *ALOX12*–3.6, +5.5; *IL-8*–8.6, +10; *CYP4A11*–1.8, +3.4. Contrary to most research showing that *PTGS1* (COX1) and *PTGS2* (COX2) are commonly co-regulated we found no correlation between the fold-change in gene expression of these two iso-enzymes in PBMCs following omega-3 supplementation (*p* = 0.7249; r = -0.1137). On the other hand, we found a significant positive correlation between *IL-8* and *PTGS2* (*p* = 0.0041, r = 0.7598, confidence interval (CI): 0.3297, 0.9287) as well as a highly significant positive correlation between *PTGS1* and *ALOX12* fold change in gene expression (p = <0.0001; r = 0.8993, CI: 0.6725, 0.9717).

There was only one statistically significant correlation between fold change in *PTGS1* and % change in ARA, EPA, or DHA, which was the % change in PE ARA (*p* = 0.0044, r = 0.7564, CI: 0.3224, 0.9276) (Fig 2). Neither EPA nor DHA from any lipid class were correlated with *PTGS1*. Statistically significant correlations between fold change in *PTGS1* and % change in oxylipin/FA ratios are shown in Table 1. All of these % changes in oxylipin/FA ratios were positively correlated with fold change in *PTGS1*. Percentage changes in the *PTGS1* products TXB_2_ (*p* = 0.0100, r = 0.7078, CI: 0.2256, 0.9115) and PGD_2_ (*p* = 0.0422, r = 0.5929, CI: 0.0288, 0.8706) were also positively correlated with fold change in *PTGS1*.

**Table 1:** Correlations between percentage change in oxylipin product/fatty acid precursor ratios and fold change in *PTGS1* gene expression in response to 6 weeks of supplementation with omega-3 fatty acids as fish oil.

% Change in Ratio*	P-value	r	Confidence Interval^
PGE_2_/FFA 20:4n6	0.0023	0.7887	0.3924–0.9380
PGD_2_/FFA 20:4n6	0.0023	0.7892	0.3935–0.9382
PGD_2_/LY 20:4n6	0.0250	0.6398	0.1042–0.8878
PGD_2_/PC 20:4n6	0.0354	0.6095	0.0547–0.8767
PGD_2_/DG 20:4n6	0.0348	0.6109	0.0570–0.8773
PGD_2_/TG 20:4n6	0.0462	0.5839	0.0151–0.8672
TXB_2_/FFA 20:4n6	0.0102	0.7063	0.2226–0.9109
TXB_2_/PC 20:4n6	0.0173	0.6691	0.1545–0.8981
TXB_2_/TG 20:4n6	0.0201	0.6576	0.1344–0.8941
TXB_2_/PE 20:4n6	0.0282	0.6296	0.0872–0.8841
TXB_2_/LY 20:4n6	0.0320	0.6186	0.0693–0.8801
TXB_2_/DG 20:4n6	0.0361	0.6077	0.0518–0.8760
TXB_2_/CE 20:4n6	0.0437	0.5894	0.0234–0.8693

*PGE2: prostaglandin E2, PGD2: prostaglandin D2, TXB2: thromboxane B2, FFA: free fatty acid, LY: lysophosphatidylcholine, PC: phosphatidylcholine, DG: diglyceride, TG: triglyceride, PE: phosphatidylethanolamine, CE: cholesterol ester

^ 95% Confidence interval for the correlation calculated using the Fisher transformation.

Just as with PTGS1, the only significant correlation between *ALOX12* and ARA, EPA, or DHA was % change in PE ARA (Fig 2). Table 2 shows the significant correlations between fold change in *ALOX12* and % changes in oxylipin/FA ratios, all of which were positive correlations just as with *PTGS1*. In this case, the ratios of both the omega-6 ARA and its corresponding *ALOX12* oxylipin product 12-HETE, and the ratios of omega-3 EPA and its corresponding product 12-HEPE were significantly correlated with *ALOX12*. However, only the % change in 12-HETE (*p* = 0.0052, r = 0.7475, CI: 0.3040, 0.9247) but not 12-HEPE (*p* = 0.2001, r = 0.3979) was significantly correlated with *ALOX12*.

**Table 2:** Correlations between percentage change in oxylipin product/fatty acid precursor ratios and fold change in *ALOX12* gene expression in response to 6 weeks of supplementation with omega-3 fatty acids as fish oil.

% Change in Ratio*	P-value	r	Confidence Interval^
12-HETE/TG 20:4n6	0.0025	0.7850	0.3842–0.9369
12-HETE/CE 20:4n6	0.0042	0.7591	0.3281–0.9285
12-HETE/FFA 20:4n6	0.0083	0.7200	0.2489–0.9156
12-HETE/PC 20:4n6	0.0103	0.7060	0.2220–0.9108
12-HETE/LY 20:4n6	0.0194	0.6606	0.1397–0.8951
12-HETE/PE 20:4n6	0.0226	0.6484	0.1186–0.8908
12-HETE/DG 20:4n6	0.0267	0.6344	0.0952–0.8858
12-HEPE/CE 20:5n3	0.0036	0.7673	0.3456–0.9312
12-HEPE/TG 20:5n3	0.0102	0.7067	0.2235–0.9111
12-HEPE/LY 20:5n3	0.0286	0.6285	0.0855–0.8837
12-HEPE/PC 20:5n3	0.0304	0.6232	0.0767–0.8817
12-HEPE/PE 20:5n3	0.0377	0.6035	0.0452–0.8745

*12-HETE: 12-hydroxyeicosatetraenoic acid, 12-HEPE: 12-hydroxyeicosapentaenoic acid, FFA: free fatty acid, LY: lysophosphatidylcholine, PC: phosphatidylcholine, DG: diglyceride, TG: triglyceride, PE: phosphatidylethanolamine

^ 95% Confidence interval for the correlation calculated using the Fischer transformation., CE: cholesterol ester

There were no statistically significant correlations between fold change in *CYP4A11* and % change in any of the FA, its product 20-HETE, or any of the 20-HETE/ARA ratios (data not shown). There were also no statistically significant correlations between fold change in *PTGS2* and % change in any of the FA, oxylipins, or oxylipin/FA ratios (data not shown). Likewise, there were no statistically significant correlations between IL-8 and any of the % changes in EPA, DHA, or ARA (data not shown).

BMI was positively correlated with both *PTGS1* (*p* = 0.0407, r = 0.5964, CI: 0.0342, 0.8719) and *ALOX12* (*p* = 0.0071, r = 0.7297, CI: 0.2680, 0.9188) fold change but not with any of the % changes in product/precursor ratios or with any of the % changes in EPA, DHA, or ARA or their oxylipin metabolites. However, there was a non-statistically significant negative correlation between % change in PE EPA and BMI (*p* = 0.0849, r = -0.5422, CI: -0.8618, -0.0855) (Fig 2), and % change in FFA EPA and BMI (*p* = 0.0502, r = -0.5755, CI: -0.8640, -0.0024). BMI was not correlated with % change in PE ARA (*p* = 0.4004, r = 0.2676) however it was negatively correlated with % change in the EPA/ARA ratio in the PE lipid class (*p* = 0.0248, r = -0.6406, CI: -0.8880, -0.1055) (Fig 2) but not any of the other lipid classes.

## Discussion

Our previously published paper describes in detail the effects of omega-3 supplementation on plasma FA and oxylipin profiles [5]. Briefly, concentrations of EPA and DHA significantly increased across lipid classes post supplementation as expected, however the magnitude of response was highly variable. For example, change in PC EPA ranged from a 104% increase to a 3,528% increase, and change in PE DHA ranged from a 4% decrease to a 187% increase. The responses in neither the FA precursors (EPA, DHA, and ARA) nor their oxylipin products were associated with age, gender, body weight, or BMI. It is possible however, that we may have seen a dose-response for EPA with a larger sample size, given that there was some evidence of a negative correlation between PE and FFA EPA and BMI in this study. Baseline mol% of ARA in PE was negatively correlated with the % change in PE DHA, suggesting that in individuals with high constitutive levels of PE ARA there is less incorporation of DHA into this lipid class. Although concentrations of ARA decreased in most subjects, the differences in ARA concentrations across lipid classes pre to post supplementation did not reach statistical significance when the Bonferroni correction was applied. Several intermediates in the desaturation and elongation pathway of ARA, including 18:3n6 and 20:3n6, decreased in response to the omega-3 supplement, suggesting that the conversion of 18:2n6 to ARA was reduced, however, the levels of ARA were preserved to a greater extent in some individuals than others.

In this study we found that healthy individuals supplemented with omega-3 FA had differential responses in *PTGS1*, *PTGS2*, *ALOX12*, *IL-8*, and *CYP4A11* gene expression in isolated PBMCs. For both *PTGS1* and *ALOX12*, % change in PE ARA was positively correlated with a change in gene expression in response to omega-3 supplementation. In those individuals for whom plasma ARA in the PE lipid class decreased in response to omega-3 intervention, there was a corresponding decrease in gene expression for *PTGS1* and *ALOX12* in PBMCs. On the other hand, changes in EPA and DHA were not correlated with either *PTGS1* or *ALOX12* gene expression changes. These findings suggest that the concentration of ARA, which is modified by omega-3 supplementation, rather than the omega-3 EPA and DHA directly, may be involved in regulating the expression of the *PTGS1* and *ALOX12* enzymes.

There were also positive correlations between fold change in *PTGS1* and *ALOX12* and % change in their respective oxylipin/FA ratios, for instance PGE_2_/FFA ARA and 12-HETE/TG ARA respectively. For *ALOX12* the ratios of 12-HEPE to its FA precursor EPA across several lipid classes were also positively correlated with fold change in gene expression. These results suggest that the activities of the enzymes decreased concomitantly in those individuals for whom gene expression decreased in response to supplementation. Together, these results suggest that those individuals who have constitutively high levels of ARA in the PE lipid class maintain their levels of *PTGS1* and *ALOX12* gene expression and activity in circulating monocytes and may not see some of the expected anti-inflammatory benefits of omega-3 supplementation. On the other hand, in individuals whose levels of PE ARA decrease there may also be a decrease in the gene expression and activity of *PTGS1* and *ALOX12*.

We found positive correlations between BMI and fold change in the expression of *PTGS1* and *ALOX12* but not between BMI and % changes in ARA, EPA, or DHA across lipid classes, or any of their oxylipin metabolites, or product/precursor ratios. Importantly, % change in PE ARA was not correlated with BMI. This suggests that the changes in PE ARA were not likely a simple dose-response to the omega-3 intervention. However, % change in the ratio EPA/ARA in the PE lipid class was significantly negatively correlated with BMI although this ratio was not associated with the fold changes in any of the 5 genes. Also, there was a non-statistically significant correlation between FFA EPA and PE EPA, and BMI. The correlations among *PTGS1* and *ALOX12* fold change and the FA, oxylipins, and FA/oxylipin ratios were on the whole stronger than the correlations with BMI, however, BMI appears to play a role in the PBMC gene expression changes in these two enzymes in response to omega-3 intervention. In previous studies, a dose-dependent effect was observed for phospholipid EPA/ARA ratios at similar dosages of omega-3 in IgA nephropathy patients (significant negative correlation between phosholipid EPA/ARA and dose of omega-3 fatty acids in g/kg body weight) [13]. However, in our study, neither the % changes in EPA nor the % change in the EPA/ARA ratio were associated with fold change in the gene expression of *PTGS1* and *ALOX12*. As mentioned earlier, it is possible that our sample size was too small to detect a direct dose-response relationship between fold change in the oxylipin metabolic genes and % change in EPA or EPA/ARA. These observations merit further study in larger cohorts.

*PTGS1* continues to be described as the constitutive isoform of the COX enzyme whereas *PTGS2* is considered the inducible form [14]. However, as early as 2000, Maldve et al. [15] demonstrated that both isoforms were inducible by ARA and its PG metabolites in murine keratinocytes. On the other hand, Vichai et al [16] showed positive feedback regulation of *PTGS2* mRNA expression and protein activity but not *PTGS1* by prostaglandin metabolites (PGE_2_ in particular) in mouse lung fibroblasts. Thus, it appears that there is tissue-dependent variability in the inducibility of the two COX isoforms.

The fold-change in *PTGS1* gene expression was highly correlated with *ALOX12* gene expression but not with *PTGS2* gene expression. On the other hand, *IL-8* and *PTGS2* were positively correlated. The co-correlation observed between *ALOX12*/*PTGS1* and *IL-8*/*PTGS2* could be explained by the fact that these genes share some common transcription factors. All 4 of these genes have AP-1 and c-Jun in common; however, *PTGS1* and *ALOX12* also have Sp1 in common whereas *PTGS2* and *IL-8* also have NF-kappa β and C/EBPα in common. It is possible that the observed associations between *PTGS1* and *ALOX12* and between *PTGS2* and *IL-8* are mediated through the induction of these transcription factors by changes in the FA composition of plasma.

The regulation of gene expression after omega-3 supplementation has been reviewed previously [17]. Omega-3 fatty acids influence the gene expression of a variety of important transcription factors involved in regulating lipid metabolism, especially peroxisome proliferator-activated receptor, sterol regulatory binding element protein, and nuclear factor kappa b. Specific downstream genes that have been shown to be down-regulated by omega-3 fatty acids include *IL-8* and *PTGS2* [17]. A recent study investigating the effects of high dose EPA + DHA supplementation on PBMC gene expression profiles in elderly individuals found down-regulation of several oxylipin metabolism genes including leukotriene A4 (LTA4) hydrolase, which converts LTA4 to leukotriene B4 (LTB4*)*, and *ALOX5* [18]. In the current study, we did not find changes in the same genes however this may be due to the fact that our study population was healthy young (age 18–55) individuals, whereas in the previously published study the subjects were elderly (age 66–80). The current study may also have been underpowered to detect these and other gene expression changes.

This study had several shortcomings. One of the challenges of testing multiple genes at once on the same plate is that each gene has different amplification characteristics. Despite the manufacturer’s attempts to standardize the plate across all of these genes on a custom plate, we still found variability in the PCR performance. We applied stringent quality control steps into our data analysis pipeline, which resulted in the exclusion of a large proportion of the 92 genes analyzed, in order to be able to analyze only those data that were most reliable. For those few genes that remained, we feel that the data are robust, given the stringent quality control screening steps. Another shortcoming of this study is that our results are not average results from technical duplicate (or triplicate) analyses. Finally, this study was a pilot study performed in a cohort of 12 healthy subjects. Therefore, it is possible that some of the correlations observed in these analyses are due to spurious correlations resulting from small sample size, and conversely, it is possible that some relationships were not detected due to the small sample size, and the results should be interpreted with this in mind. Larger studies need to be performed to confirm these findings and further explore the nature of the metabolic phenotype differences among those individuals who appear to be responders compared to those who appear to be less responsive to omega-3 supplementation.

## Conclusions

The aim of this study was to determine whether the observed inter-individual variability in lipid response to omega-3 FA intervention may be due to an underlying variability in the regulation of gene transcription of key genes involved in the production of inflammatory lipid mediators. The results from this pilot study showed that the regulation of important oxylipin metabolic genes (e.g. *PTGS1*, *ALOX12*) in PBMCs in response to omega-3 supplementation were associated with the extent of change in ARA concentrations, particularly in the PE lipid class. Plasma concentration of PE ARA may be a biomarker of differential phenotypes in omega-3 response. Individuals with larger decreases in PE ARA after supplementation with 6 g/d fish oil had a larger decrease in the expression of *PTGS1* and *ALOX12*. These results suggest that the individual variability in response to omega-3 intervention in PBMC gene expression may be dependent on the indirect effects of the omega-3 FA on ARA concentrations rather than the extent of change to omega-3 concentrations themselves. Our results provide preliminary in vivo evidence that plasma oxylipins may be products of PBMC metabolism and that plasma FA and oxylipins are associated with PBMC gene expression in response to omega-3 intervention. The results of this pilot study indicate that the gene expression of key lipid metabolic genes in PBMCs varies with changes in plasma FA and oxylipin composition in response to omega-3 supplementation to different degrees in different individuals.

## Supporting Information

S1 Protocol Approved Study Protocol. (PDF)

S1 Table Age, gender, height, weight, and body mass index (BMI), of the 12 subjects in this study at baseline. (DOCX)

S1 TREND Checklist TREND Statement Checklist. (PDF)

## Abbreviations

FA: fatty acids
PBMCs: peripheral blood mononuclear cells
EPA: eicosapentaenoic acid
DHA: docosahexaenoic acid
qPCR: quantitative polymerase chain reaction
PTGS1: prostaglandin-endoperoxide synthase 1
PTGS2: prostaglandin-endoperoxide synthase 2
ALOX12: 12-lipoxygenase
ARA: arachidonic acid
CYP: cytochrome P450
IL-8: interleukin 8
PUFA: polyunsaturated fatty acids
COX: cyclooxygenase
LOX: lipoxygenase
PGE2: prostaglandin E2
TXB2: thromboxane B2
PGD3: prostaglandin D3
PGE3: prostaglandin E3
5-LOX: 5-lipoxygenase
HETE: hydroxyeicosatetraenoic acid
HEPE: hydroxypentaenoic acid
LA: linoleic acid
BMI: body mass index
RQI: RNA quality indicator
CE: cholesterol ester
DG: diglyceride
FFA: free fatty acid
LY: lyso phosphatidylcholine
PC: phosphatidylcholine
PE: phosphatidylethanolamine
TG: triglyceride
SCD: stearoyl-CoA-desaturase
SPE: solid phase extraction
MRM: multiple reaction monitoring
LOQ: limit of quantification
LTA4: leukotriene A4
LTB4: leukotriene B4
