# Supplementary material for: Changes in PTGS1 and ALOX12 Gene Expression in Peripheral Blood Mononuclear Cells Are Associated with Changes in Arachidonic Acid, Oxylipins, and Oxylipin/Fatty Acid Ratios in Response to Omega-3 Fatty Acid Supplementation
Source: PLoS One. 2015 Dec 16;10(12):e0144996. doi: 10.1371/journal.pone.0144996 (PMC4681469; doi:10.1371/journal.pone.0144996)
Supplement: S1 Protocol — (PDF) [file pone.0144996.s001.pdf]

S1 Text. Approved Study Protocol

INSTITUTIONAL REVIEW BOARD
University of California, Davis

PROTECTION OF HUMAN SUBJECTS – DECLARATION / ASSURANCE OF IRB APPROVAL

**Principal Investigator**<br>Bruce Hammock	**Protocol No.**<br>200715313-1	**Approval Date**<br>05/03/07	**Expiration Date**<br>05/03/08	**Risk Level**<br>Minimal Risk
**PI Department**<br>ENTOMOLOGY	**Sponsor Name**<br>Superfund Training Fellowship	**Level of Review**<br>Expedited	**Expedited Category**<br>2a ,3 ,7	**Status**<br>New

This Assurance, on file with the Department of Health and Human Services, covers this activity:

FWA No: 00004557 **Expiration Date**: January 18, 2010 **IORG**: 0000251

The following research study has been reviewed by the IRB in accordance with the Common Rule and any other governing regulations:
Development Of A Metabolic Assessment Tool For Chronic Kidney Disease

The above referenced activity has been determined to meet the definition of human subjects research as defined by Federal Regulations and UC Davis IRB Policy. As principal investigator for a study involving human subjects, you assume certain responsibilities, specifically:

1. You will conduct the study according to the protocol approved by the IRB. As the PI you will be accountable for your own research and the protection of human subjects. You will ensure, at all times, that you have the appropriate resources and facilities to conduct this study. You will ensure that all research personnel involved in the conduct of the study have been appropriately trained on the protection of human subjects, in addition to the study procedures.
2. Any unanticipated problems involving risks to participants or others will be reported to the IRB in accordance with IRB policy. Changes in approved research initiated without IRB approval to eliminate apparent immediate hazards to the participant, are to be reported to the IRB in accordance with the policy "Reporting of Unanticipated Problems Involving Risks to Participants or Others."
3. Any changes in your research plan must be submitted to the IRB for review and approval prior to implementation of the change. Proposed changes in approved research cannot be initiated without IRB approval, except when necessary to eliminate apparent immediate hazards to participants.
4. Your protocol must be renewed prior to expiration of the study. Although a courtesy renewal notice will be issued to you four months prior to expiration, should you fail to receive this notice it is your responsibility to contact the IRB Administration for a duplicate copy. Failure to submit renewal documents to the IRB Administration by the Administrative Due Date may result in termination of the study by the IRB.
5. Advertisements for the recruitment of subjects must be approved by the IRB prior to implementation.
6. If you plan to collect protected health information, you are required to comply with HIPAA requirements.
7. Studies conducted at the CCRC must be reviewed and approved by the VA Research & Development Committee prior to initiation of the study. Contact the VA R&D Committee for submission requirements.
8. Should your study involve the use of investigational drugs, you are required to provide a complete copy of the approved protocol to the Investigational Drug Service Pharmacy.

: this is a Clinical Study, the Hospital Health System requires that:

- A complete copy of the IRB approved Description of Study and signed Consent Form be placed in the patient's medical record. Ensure that you have swiped the patient's name plate card or printed the patient's name at the top of page 1 of the consent document. Medical procedures should be documented in the patient's medical record.
- All investigational drugs be distributed through the UCDMC Pharmacy. A copy of the signed consent form must be submitted to the Pharmacy if investigational drugs are dispensed through the Outpatient Pharmacy.
- If the study involves radiation use, a copy of the IRB approved consent form be sent to: RUC, Health Physics, 2500 FSSB.

**Name and Address of Institution:**<br>University of California, Davis<br>IRB Administration<br>2921 Stockton Blvd., Suite 1400, Rm. 1429<br>Sacramento, CA 95817	**Signature : The IRB Chair/Designee signing below certifies that the information provided above is correct and that, as required, future reviews will be performed until study closure and certification will be provided.**<br>Jeanette R. Pleasure, M.D.
**Institutional Administrator:**<br>Anthony Perez, JD, Director, IRB Administration<br>Anthony.Perez@ucdmc.ucdavis.edu	**Name:**<br>Jeanette Pleasure MD
**Phone No.** (916) 703-9151	**Title:**<br>Scientific Member
**Fax No.** (916) 703-9160	**Date:**<br>05/15/2007

Std. 05/15/2007

DESCRIPTION OF STUDY

Please address each section carefully and in detail. Failure to do so will delay the review of your study.

PURPOSE AND PROCEDURES:

1. Describe the study format and whether it is single or multi-center; industry-sponsored or investigator initiated; and the funding source.

This is a multi-center, investigator-initiated **study examining the variation between individuals in blood lipid metabolites, cytokines, gene expression, metabolomic fingerprint, and blood and urine eicosanoid levels, and the changes in these levels in response to omega-3 fatty acid (**w**3FA) supplementation in patients with immunoglobulin A nephropathy (IgAN) compared with healthy controls. The funding source is a pilot grant from the Center for Human Nutrition Research (CHNR).**

2. Briefly describe the specific aims of the study, research methods and procedures.

It is unclear to what extent blood lipid levels, cytokines, gene expression, metabolomic fingerprint, and blood and urine eicosanoid levels vary between individuals, and it is also unclear how they change in response to short-term w**3FA or fish oil supplementation. It is necessary to establish the means and standard deviations in the levels of these metabolites in the healthy population, in order to better understand the mechanisms related to lipid metabolism of metabolic diseases that are modified by** w**3FA. Specifically, the project will examine the differences between healthy controls and patients with immunoglobulin A nephropathy (IgAN) before and after** w**3FA supplementation. The IgAN patient samples will be obtained from collaborators from a previously completed project. These samples have been stored in a freezer and are awaiting analysis. The portion of the project involving the healthy controls remains to be carried out.**

This project will resolve the following **specific aims**:

- Establish preliminary estimates of means and standard deviations in the levels of w3FA within different lipid classes in the blood, the levels of inflammatory cytokines, gene expression, metabolomic fingerprint, and the extent of variation between healthy individuals compared with IgAN patients in these variables before and after w3FA/fish oil supplementation.
- Determine preliminary estimates of means and standard deviations in the levels of eicosanoids in the blood and urine of healthy individuals compared with IgAN patients before and after w3FA/fish oil supplementation.
- Develop methods and preparative protocols for the analysis of w3FA-derived eicosanoid metabolites in the blood and urine using samples from healthy individuals and IgAN patients.

The **research methods and procedures** that will be used for this study involve the following:

- ~~Thirty healthy volunteers willing and able to take 6 g of fish oil for 6 weeks will be recruited from the general UC Davis population including students, faculty, and staff. The volunteers will be recruited by personal communication at seminars and other school-related activities as well as flyers that will be posted on campus.~~
- ~~The volunteers will be contacted and a mutually agreed-upon date will be scheduled for a brief meeting during which time subjects will receive a copy of the consent form to read over, as well as the first morning urine collection tube. This will be followed by a mutually agreed-upon date for~~

~~scheduled for a brief meeting during which time subjects will receive a copy of the consent form to read over, as well as the first morning urine collection tube. This will be followed by a mutually agreed-upon date for the first blood draw and urine collection. The researcher will contact the volunteer the day before the first scheduled study date to confirm and remind the volunteer about fasting, about collecting the first morning urine void, and the time and location of the study.~~

- The participants will come to the Ragle Human Nutrition Center (1283 Academic Surge) between 7 and 9 am after an overnight fast, at which point they will be weighed (clothed but with no shoes) on a digital scale, as well as sign a consent form and discuss all their rights regarding the study. A registered phlebotomist will take 20 mL of blood by venipuncture for their pre-supplementation blood draw. Volunteers will turn in their first morning urine sample at this time.
- Immediately after blood collection, the researcher will separate the red blood cells and peripheral blood mononuclear cells (PBMC) from the plasma, aliquot the samples, and then store them in a -70°C freezer for future molecular and genetic analyses.
- Lipid analysis will be performed at Lipomics Technologies Inc. in West Sacramento, using published methods for quantitative lipid analysis (1). Briefly, lipids will be extracted with chloroform/methanol, followed by derivatization with methanolic HCl, separation of lipid classes with liquid chromatography, and finally fatty acid identification and quantitation with gas chromatography.
- Eicosanoid analyses will be performed in the Hammock Lab using published methods for quantitative eicosanoid analysis (2).
- Untargeted metabolites in the mass range of 100 – 1600 will be analyzed by liquid chromatography coupled to mass spectrometry in the Hammock Lab according to published methods for global metabolomic fingerprinting (3).
- One aliquot of PBMC will be stored for future gene expression analysis and DNA will be isolated from a second aliquot and quantified using Qiagen DNA isolation and Picogreen quantitation kits, respectively, according to the manufacturer's instructions. Gene expression and genotyping analyses will be performed using Taqman-based assays (4).
- Cytokine analyses will be performed using multiplexed cytokine analysis as previously described (5).
- Data analysis will be performed using standard statistical techniques using JMP software and specialized bioinformatic tools developed at Lipomics for lipidomic data analysis using specially developed software.

3. Address if therapeutically removed tissue will be collected, what types, and for what purposes.

No **tissues** will be removed for these analyses.

4. Specify the nature, frequency and duration of tests, if any.

The blood draws and urine collections will be performed once before and once after 6 weeks of supplementation with fish oil. The volunteers will come in on three occasions to the Ragle Center. On the first visit, the volunteers will meet with the researcher to go over the consent form, make an appointment for their blood draw visits, and obtain their first morning urine collection equipment as well as instructions on how to use it. On the scheduled first blood draw visit, they will have a fasting blood draw taken first thing in the morning, and at the same time bring in their first morning urine sample and obtain their 6 weeks of fish oil capsules. After 6 weeks the volunteers will return for the post blood draw visit, on which occasion they will again bring in a first morning urine sample and have a fasting blood draw taken. These 3 occasions are the only times the volunteers will need to come to the Center. They will take the capsules each day at their convenience at home/work.

sample and have a fasting blood draw taken. These [illegible] occasions are the only times the volunteers will need to come to the Center. They will take the capsules each day at their convenience at home/work.

5. If blood samples will be collected, identify in what manner and the maximum amount that will be collected over any 6 week period (if subjects are co-enrolled in other research studies, the volume of blood from the other study should also be included):

X venipuncture ___venous catheter ___arterial puncture ___arterial catheter ___cutaneous

The blood samples will be collected by **venipuncture**, and the **maximum amount** that will be collected at each session is 100 mL, and over 6 weeks is 200 mL.

6. Any additional procedures (noninvasive) involved in this study activity must be described.

There will be no **additional procedures** involved in this study.

7. If the study involves incomplete disclosure, provide the rationale.

There will be no **incomplete disclosure** involved in this study. Volunteers will be fully informed of the analyses that will be performed with their samples, of the aims of the study, and of all the procedures that will be performed during the study.

8. If this activity will be utilizing existing data, specify the source and how the data will be retrieved, reviewed, coded and stored.

Existing data and samples will be collected from our collaborator for the IgAN patients. The samples are currently being shipped to us from our collaborator. The samples and data will arrive with no identifiers and will be coded with a randomly-generated ID number corresponding to each volunteer.

There will be no existing data collected from the healthy control volunteers. They will only be asked to fill out a brief questionnaire for their age, gender, whether they are pregnant, whether they are currently using any medications and if so which ones, and whether they consider themselves to be healthy. The volunteers will also be weighed on a digital scale clothed but without shoes. They will provide 2 fasting blood samples and 2 first morning urine samples, once before and 6 weeks after supplementation with fish oil. All data will be recorded in a digital file (Excel worksheet), which will be backed up on a disc.

Each volunteer will be identified by a random, computer-generated 5-digit number. The file will be stored without any identifiers (the subjects' names or contact information will not be recorded) on the Hammock lab backup server. Contact information for the volunteers, so that a reminder e-mail or phone call can be made, will be kept in a logbook separate from the digital file containing volunteer information, and will not be traceable to the samples or to the file.

9. Address the location and duration of the study including follow-up period.

Volunteers will come to the Ragle Human Nutrition Center at 1283 Academic Surge on the UC Davis campus, and they will be there for about 40 minutes in the morning between 7 and 9 am once before and then again 6 weeks after supplementation with fish oil on the blood draw days, as well as for a brief 15 minute meeting prior to the blood draw visits.

10. Clarify how you plan to monitor data to ensure subject safety.

Only healthy, adult, non-pregnant volunteers weighing at least 110 pounds will be recruited for this study. Only limited medical history and other personal information will be collected from volunteers to determine their health status. Specifically, the volunteers will be asked some questions to ascertain whether they are healthy and fit to go through the study protocol with no danger to their health and welfare. The questions are: 1) whether they are currently using any prescription or non-prescription medications, and if so which ones; 2) whether they have been diagnosed by their physician with a disease, and if so which one(s); 3) whether they currently have any form of

health and welfare. The questions are: 1) whether they are currently using any prescription or non-prescription medications, and if so which ones; 2) whether they have been diagnosed by their physician with a disease, and if so which one(s); 3) whether they currently have any form of anemia; 4) whether they have any other health conditions or concerns that might preclude them from participating in this study, and if so to list these; and 5) whether they have been ill with a cold or flu or had a major injury in the last 2-4 weeks. Subjects will be excluded if thy have been diagnosed with a disease, if they are pregnant or nursing or if they suspect they might be pregnant, if they have some form of anemia or are not sure whether they have anemia, if they had an illness, infection, or injury within the last 2-4 weeks, and if they are taking any blood thinners or other anticoagulants. Volunteers will also be excluded if they are taking medications that alter lipid metabolism such as statins (HMG CoA reductase inhibitors), any kind of steroids, and PPAR agonists. However, we do not expect to encounter individuals who have not been diagnosed with a disease but who are taking any such medications. Volunteers will be excluded if they are taking over-the-counter allergy medication and NSAIDS, however, if they are willing to discontinue these for the period of the study they will not be excluded. In the unlikely event that the fish oil supplement causes side effects volunteers will be instructed to contact their doctor immediately, and also to contact the investigators by phone, or e-mail as soon as possible. No other data monitoring is planned for this study.

11. Address whether you have the appropriate resources (study personnel and facilities) to conduct this study.

The **appropriate resources** that will be needed and that are available to execute this study include the following:

- a phlebotomist to draw the blood,
- a researcher to recruit/prepare/guide the volunteers,
- a researcher to process the blood samples after collection,
- a researcher to collect and distribute the first morning urine collection supplies and fish oil supplements to the volunteers
- a researcher to process the urine containers
- supplies for blood collection: gloves, blood collection tubes, pipettes, pipette tips, storage tubes, lab coat, bags and boxes for sample transportation between the Ragle Center, the German Lab, and the Steinberg Lab, biohazard bags and disposal bins
- supplies for first morning urine collection and processing: cups, pipettes, pipette tips, containers
- supplies for lipid analysis: gloves, face masks, TLC plates, reagents/solutions, pipettes, pipette tips, storage tubes, transfer pipettes, GC tubes, GC columns,
- supplies for lipoprotein separation: gloves, face masks, pipettes and pipette tips, transfer pipettes, density solutions, centrifuge tubes, centrifuge tube tops,
- equipment: for blood processing – desktop centrifuge and biohazard hood, ice machine; for lipid analysis – GC equipped with columns; for lipoprotein separation – centrifuge, biohazard hood.
- equipment for cytokine analysis, gene expression analysis, and metabolomic fingerprinting: analysis kits, LC/MS equipped with columns, pipettes, storage tubes, reagents, chemicals

12. Describe the role of each <u>key</u> member of your study personnel.

- Angela M. Zivkovic: recruitment and organization of volunteers, preparation and management of consent forms and other materials, blood processing, data analysis, urine processing
- Christine M. Hegedus: vounteer recruitment and consenting, blood processing, urine processing, cytokine analysis, genotyping and gene expression analysis, data analysis
- Malin L. Nording: vounteer recruitment and consenting, blood processing

processing, urine processing, cytokine analysis, genotyping and gene expression analysis, data analysis
- Malin L. Nording: vounteer recruitment and consenting, blood processing, urine processing, metabolomic fingerprinting, data analysis
- Katrin Georgi: eicosanoid analysis and technical support
- undergraduate student interns: to assist Angela, Christine, Malin, and Katrin with duties above
- A phlebotomist at the Ragle Center will collect the blood
- Bruce D. Hammock: oversight

SUBJECT SELECTION:

1. Identify the subject population.

The **subject population** will consist of 30 healthy non-pregnant adults 18-65 years of age, weighing at least 110 pounds. The subjects will be individuals who have not been diagnosed with any disease, who are not taking any prescription medications that affect lipid metabolism or that are anticoagulants/blood thinners, who do not have any form of anemia, and who have not had any illness, infection, or injury on the last 2-4 weeks.

2. Address how subjects will be recruited: ___direct person to person solicitation, ___by telephone, ___letter, ___advertisement, ___press release, ___notices, ___other. Provide the text.

The **subjects will be recruited** with flyers posted around the UC Davis campus. The subjects will include students, faculty, and staff. The subjects will be told the following:

"We are looking for volunteers to participate in a fish oil supplementation study. You will need to come in once before the supplementation for a blood draw and a first morning urine collection. You will take 6 g of fish oil every day for 6 weeks, and not eat any seafood, or seaweed during that time. Then you will come back for another blood draw and first morning urine collection. You would need to be a healthy adult between the ages of 18 and 65, weigh at least 110 pounds, not be taking any medications, not pregnant, and not diagnosed with any diseases in order to participate in the study. If you qualify, would you be interested in participating in this study?"

3. State from where subjects will be recruited, when and how many.

The subjects will be recruited **from the general population of the UC Davis campus, and will include students, faculty, and staff. Thirty volunteers will be recruited over a 6-month period between March and August of 2008.**

4. Specify the age of the research subjects.

The subjects' **age** will be between 18 and 65 years old.

5. List all criteria for including and excluding subjects.

a. Inclusion criteria:

i. Weight at least 110 pounds
ii. Adult (aged 18-65 years old)
iii. Disclose which medications currently taking
iiii. Able to come to the Ragle Center at the designated times
v. Able to give blood
vi. Able to take 6 g fish oil per day for 6 weeks
vii. Able to carry out first morning urine collection
[illegible]

vii. Able to carry out first morning urine collection

viii. Able to stop or avoid taking NSAIDS and allergy medications for 6 weeks

ix. Able to stop or avoid eating seafood and seaweed for 6 weeks

b. Exclusion criteria:

i. Pregnant or nursing (or unsure if pregnant)

ii. Diagnosed with a disease by their physician

iii. Currently taking prescription medications that alter lipid metabolism (such as HMG CoA reductase inhibitors, PPAR agonists, steroids) and/or anti-coagulants

iiii. Currently has some form of anemia (or unsure)

v. Has an existing health condition or concern

vi. Unable to stop or avoid taking NSAIDS and allergy medications for 6 weeks

vii. Unable to stop or avoid eating seafood o seaweed for 6 weeks

viii. Unable to give blood or do first morning urine collection

ix. Recently recovering from a major injury, infection, or illness (in the last 2-4 weeks)

6. If women and minorities are excluded, provide rationale for such exclusion.

Women and minorities will not be excluded from this study.

7. Attach the translated documents for subjects whose primary language is not English.

Subjects whose primary language is not English will not be explicitly recruited for this study.

<u>SPECIAL/VULNERABLE POPULATION (if applicable):</u>

Not applicable

***<u>RISKS</u>*:**

1. Address whether there is a possibility of physical, psychological, social or legal injury from participation in this study and assess the likelihood and seriousness of those risks.

The **risks** of drawing blood from a vein include discomfort at the site of puncture; possible bruising and swelling around the puncture site; rarely an infection; and, uncommonly, faintness from the procedure. These risks are considered to be slight for healthy people. There may be a slight risk of gastrointestinal discomfort resulting from fish oil supplementation.

Fish oil and w3FA are considered to be safe and have minimal side effects even at very high doses (20 g per day), as shown in several studies, even in vulnerable populations such as individuals who recently underwent gastric surgery and patients with end stage renal disease (6-9). The daily dose of fish oil that will be administered as part of this study is similar to consuming a 9-ounce fillet of wild Coho salmon, which provides 19 g fat, and 5 g w3FA with 2.1 g DHA and 1.4 g EPA. In comparison, the dosage of fish oil that will be consumed by the subjects will provide 6 g fish oil, and 3.5 g w3FA with 1.5 g DHA and 2 g EPA.

<u>The methods used in this study are the least risky, and most widely accepted method for drawing blood from healthy adult humans, therefore, no other methods were considered. Omacor is refined fish oil which provides a higher dosage than fish oil of EPA and DHA as</u>

The methods used in this study are the least risky, and most widely accepted method for drawing blood from healthy adult humans, therefore, no other methods were considered. Omacor is refined fish oil which provides a higher dosage than fish oil of EPA and DHA as ethyl esters, and was the supplement administered to the IgAN patients. However, Omacor is only available by prescription, and therefore, fish oil which is available over-the-counter and which will provide comparable amounts of w3FA **and EPA/DHA, will be used for the control subjects.**

2. If the methods of research create potential risks, describe other methods, if any, that were considered and why they will not be used.

The **methods** used in this study are the least risky, and most widely accepted method for drawing blood from healthy adult humans, therefore, no other methods were considered. Enteric-coated fish oil was selected due to its diminished potential for gastrointestinal side-effects. Omacor is refined fish oil which provides a higher dosage than fish oil of EPA and DHA as ethyl esters, and was the supplement administered to the IgAN patients. However, Omacor is only available by prescription, and therefore, fish oil which is available over-the-counter and which will provide comparable amounts of w3FA and EPA/DHA, will be used for the control subjects.

3. Identify your plan for protecting subject privacy and confidentiality.

The subject’s name and contact information will be kept separate from his/her blood analysis data. Contact information will be kept only for the purposes of coordinating recruitment, and will be kept in a logbook separate from the digital file containing the personal information. Blood samples and all associated data derived from them will be anonymous and coded by a randomly-generated ID number.

4. Explain your plan for reporting adverse and serious adverse events to the IRB.

A report detailing the events and symptoms that resulted will be written and submitted as quickly as possible to the IRB office by fax, regular mail, and/or e-mail via the Adverse Event Report Form. However, no adverse and serious events resulting from the consumption of 6 g/d fish oil or from simple blood draws and urine collections are anticipated in healthy adults.

5. If the study involves the use of placebo, justify why this is appropriate.

This study does not involve the use of a placebo**.**

BENEFITS:

1. Address if there is a benefit to individual subjects or to the particular group or class.

The **benefits** of this study **to individual subjects** involve participation in research to improve the assessment of metabolic health.

2. Address if there is no direct benefit to the subject.

The **direct benefit to the individual subject** is 6 weeks of free fish oil supplement.

RISK-BENEFIT RATIO:

1. Address whether the risks to subjects are reasonable in relation to the benefits (note: do not state that the benefits outweigh the risks. Rather, construct a summary assessment of the relative risks (physical, psychological, economical, and legal) to participants versus the potential benefits to participants and society).

The **relative risks** of injury resulting from standard blood draw procedure (discomfort, bruising, infection at the site of puncture, possible faintness from blood loss, and possible gastrointestinal discomfort from fish oil supplement are minor **in relation to** the direct benefit of 6 weeks of free fish oil supplementation, and the **potential benefits** of improving assessment and analysis techniques that will result from these investigations.

COSTS/COMPENSATION TO SUBJECTS

that will result from these investigations.

***COSTS/COMPENSATION TO SUBJECTS*:**

1. If the study involves the possibility of added expenses to the subject or to a third party, such as an insurer (e.g., longer hospitalization, extra laboratory tests, travel) address the magnitude of those expenses and how this is justified.

There are **no added expenses** to the subject or to any third party associated with this study.

2. Describe the amount and type of compensation that will be paid to subjects and how that compensation will be staged/pro-rated.

The **compensation** to the subjects will be 6 weeks of free fish oil supplements.

***DISCLOSURE OF PERSONAL AND FINANCIAL INTEREST*:**

1. Disclose any personal and financial interest in the research as well as the extent of personal and financial interest in the sponsor.

No personal and financial interest is associated with this research.

WAIVER OF INFORMED CONSENT (if applicable):

Not applicable.

REFERENCES

1. Watkins SM, Reifsnyder PR, Pan HJ, German JB, Leiter EH. Lipid metabolome-wide effects of the PPARgamma agonist rosiglitazone. J Lipid Res 2002;43:1809-17.
2. Schmelzer KR, Wheelock AM, Dettmer K, Morin D, Hammock BD. The role of inflammatory mediators in the synergistic toxicity of ozone and 1-nitronaphthalene in rat airways. Environ Health Perspect 2006;114:1354-60.
3. Cubbon S, Bradbury T, Wilson J, Thomas-Oates J. Hydrophilic interaction chromatography for mass spectrometric metabonomic studies of urine. Anal Chem 2007;79:8911-8.
4. Hegedus CM, Skibola CF, Bracci P, Holly EA, Smith MT. Screening the human serum proteome for genotype-phenotype associations: an analysis of the IL6 -174G>C polymorphism. Proteomics 2007;7:548-57.
5. Penolazzi L, Lambertini E, Tavanti E, et al. Evaluation of chemokine and cytokine profiles in osteoblast progenitors from umbilical cord blood stem cells by BIO-PLEX technology. Cell Biol Int 2008;32:320-5.
6. Donadio JV, Jr., Grande JP, Bergstralh EJ, Dart RA, Larson TS, Spencer DC. The long-term outcome of patients with IgA nephropathy treated with fish oil in a controlled trial. Mayo Nephrology Collaborative Group. J Am Soc Nephrol 1999;10:1772-7.
7. Donadio JV, Jr., Larson TS, Bergstralh EJ, Grande JP. A randomized trial of high-dose compared with low-dose omega-3 fatty acids in severe IgA nephropathy. J Am Soc Nephrol 2001;12:791-9.
8. Harris WS, Ginsberg HN, Arunakul N, et al. Safety and efficacy of Omacor in severe hypertriglyceridemia. J Cardiovasc Risk 1997;4:385-91.
9. Hogg RJ. A randomized, placebo-controlled, multicenter trial evaluating alternate-day prednisone and fish oil supplements in young patients with immunoglobulin A nephropathy. Scientific Planning Committee of the IgA Nephropathy Study. Am J Kidney Dis 1995;26:792-6.
