# Supplementary material for: Changes in PTGS1 and ALOX12 Gene Expression in Peripheral Blood Mononuclear Cells Are Associated with Changes in Arachidonic Acid, Oxylipins, and Oxylipin/Fatty Acid Ratios in Response to Omega-3 Fatty Acid Supplementation
Source: PLoS One. 2015 Dec 16;10(12):e0144996. doi: 10.1371/journal.pone.0144996 (PMC4681469; doi:10.1371/journal.pone.0144996)
Supplement: S1 Table — (DOCX) [file pone.0144996.s002.docx]

**S1 Table.** Age, gender, height, weight, and body mass index (BMI) of the 12 subjects in this study at baseline.

| **Subject ID** | **Age** | **Gender** | **Height (m)** | **Weight (kg)** | **BMI (kg/m^2^)** |
| --- | --- | --- | --- | --- | --- |
| **202** | 26 | F | 1.75 | 66.1 | 22 |
| **203** | 31 | F | 1.57 | 49.0 | 20 |
| **206** | 33 | F | 1.63 | 69.2 | 26 |
| **207** | 32 | F | 1.73 | 64.0 | 21 |
| **212** | 26 | M | 1.75 | 79.5 | 26 |
| **214** | 21 | F | 1.63 | 54.5 | 21 |
| **218** | 35 | M | 1.83 | 94.4 | 28 |
| **220** | 55 | M | 1.70 | 65.0 | 22 |
| **222** | 28 | F | 1.60 | 52.6 | 21 |
| **225** | 34 | M | 1.91 | 96.8 | 27 |
| **227** | 32 | F | 1.55 | 52.6 | 22 |
| **231** | 28 | F | 1.63 | 50.3 | 19 |
| **Mean +/- SD** | 32 +/- 8 | – | 1.69 +/- 0.11 | 67 +/- 17 | 23 +/- 3 |
